# Immunomodulatory Effects of Tryptophan Metabolism in the Glioma Tumor Microenvironment

**DOI:** 10.3389/fimmu.2021.730289

**Published:** 2021-10-01

**Authors:** Yang Xu, Huikai Zhang, Qian Sun, Rongxin Geng, Fanen Yuan, Baohui Liu, Qianxue Chen

**Affiliations:** Department of Neurosurgery, Renmin Hospital of Wuhan University, Wuhan, China

**Keywords:** glioma, tryptophan, metabolism, immunomodulation, tumor microenvironment

## Abstract

Gliomas are the most common primary malignant tumor in adults’ central nervous system. While current research on glioma treatment is advancing rapidly, there is still no breakthrough in long-term treatment. Abnormalities in the immune regulatory mechanism in the tumor microenvironment are essential to tumor cell survival. The alteration of amino acid metabolism is considered a sign of tumor cells, significantly impacting tumor cells and immune regulation mechanisms in the tumor microenvironment. Despite the fact that the metabolism of tryptophan in tumors is currently discussed in the literature, we herein focused on reviewing the immune regulation of tryptophan metabolism in the tumor microenvironment of gliomas and analyzed possible immune targets. The objective is to identify potential targets for the treatment of glioma and improve the efficiency of immunotherapy.

## Introduction

Gliomas are the most common primary malignant tumor among brain and other CNS tumors (24.1% of all tumors). Additionally, glioblastoma accounts for 60% of gliomas. Although standard treatment, including surgical resection, targeted radiation therapy, chemotherapy treatment, has significantly progressed (1), the median survival for glioblastoma for all patients (regardless of treatment) remained 14-15 months (2). Immunotherapy and tumor-treating fields (TTFields) (3, 4) have swiftly developed in recent years, but there is yet to be any breakthrough. Meanwhile, more and more investigations have revealed novel biomolecular insights that focus on the metabolism of cells to explore the malignant phenotype of gliomas (5, 6). Indeed, the tumor microenvironment of glioma is emerging as a critical regulator of cancer progression in the immune-suppressive aspect (7). These highly and complex netting metabolic pathways, which exist in both the tumor cell and the microenvironment, are surfacing throughout onco-metabolomics in gliomas.

Amino acid metabolism emerges as an essential role in the metabolic reprogramming of cancer cells. A previous study has constructed an amino acid-related risk signature for gliomas, which could predict patients’ survival and clinical features (8). Amino acids and their derivatives can not only regulate cancer cells but also modulate the surrounding microenvironment, which enhances malignancy and immunosuppression (9). For instance, Kynurenine, the catabolic product of tryptophan, induces the invasion of cancer cells and immunosuppression of the tumor microenvironment (10, 11) by binding to transcription factor aryl hydrocarbon receptor (12–14). Moreover, the activation of AHR hampers the performance of macrophages and T cells, which play an antitumoral role (10, 14). Thus, the metabolism of amino acids is diverse in tumors and has a crucial role not only in the biological process of tumor cells but also in the tumor microenvironment, particularly in the modulation of the immune system. These indicate that a better comprehension of amino acid metabolism will provide potentially efficient targets for glioma treatment (15, 16).

Herein, we focused on reviewing how the immune mechanism in the glioma tumor microenvironment is affected by tryptophan metabolism. The aim is to provide potential targets for the treatment of glioma and improve the efficacy of immunotherapy.

## The Immune Microenvironment of Glioma

The brain tumor microenvironment is composed of tumor populations, interstitial cells and immune cells. The majority of immune cells in gliomas, including GBM, comprises a vast diversity of myeloid cells, which include bone marrow-derived macrophages (BMDMs), microglia, myeloid-derived suppressor cells (MDSCs), dendritic cells and neutrophils (17). Glioma-associated macrophages (GAMs) are the primary immune component of brain gliomas, which consisting of a mixture of bone marrow-derived macrophages (BMDMs) and resident macrophages called microglia (MG) (18). These cells are recruited into the glioma environment and can release a large number of growth factors and cytokines to influence tumor proliferation, migration and so on.

Recruitment of GAMs into the microenvironment is driven by multiple chemokines secreted by gliomas, including monocyte chemotactic protein (MCP)-1 (19), MCP-3 (20), TME motif chemokine 12(CXCL12) (21), colony-stimulating factor (CSF-1) (22), glial cell-derived neurotrophic factor (GDNF) and granulocyte-macrophage colony-stimulating factors (23). These factors are expressed differently in different glioma types and may guide the variations in immune infiltration. Studies have found that the degree of GAMs invasion is correlated with tumor grade (24, 25), and differences in GAMs were found in the microenvironment of gliomas with different isocitrate dehydrogenase 1/2 (IDH1/2) somatic mutation types (26). IDH1/2 mutation-induced methylation suppresses the recruitment of TAM and induces an immune response in gliomas. Compared to IDH wild type, patients with IDH mutations have lower immune infiltration and better prognosis. Glioblastoma can be classified into classical (CL), mesenchymal (MES), neural (N), and neurogenic (PN) tumors based on unique transcriptome characteristics (27). Compared with non-MES tumors, several cell types were increased in MES-GBM, including CD4+T cells, type 2 polarized macrophages, and neutrophils (28). MES-GBM was associated with vascular remodeling and immune cell aggregation, while CL/PN-GBM was linked to reduced immune cell infiltration and better prognosis (29).

Besides GAMs, several other immune cells have been identified in the glioma parenchyma, although the proportion is very low. In fact, T cells account for the majority of lymphocytes in glioma. CD8+ cytotoxic T cells are cellular immune effectors that are essential for killing tumor cells, but they are only sparsely distributed in the GBM parenchyma (30). These cells may not exert significant effector responses, and their function is impaired by immunosuppressive factors derived from myeloid cells (such as GAMs) (31). We speculate that a higher level of GAMs may hinder the infiltration of effector T cells. Moreover, regulatory T cells (Tregs) are also present in the glioma parenchyma. These cells actually possess immunosuppressive functions and are thought to inhibit anti-tumor immunity in various solid tumors such as ovarian, breast, and pancreatic cancers (32).

Glioma cells escape the immune system by inhibiting immune mechanisms in the microenvironment. Furthermore, glioma releases immunosuppressive and tumor-support factors to the microenvironment, resulting in proliferation, invasion, and immune escape (33).

## Tryptophan Metabolism

Tryptophan (Trp) is an essential amino acid for animals and humans, obtained mainly from the diet. Protein in food is digested in the small intestine to release Trp, which is subsequently absorbed into the bloodstream through the intestine (34, 35). The most significant function of free Trp is its contribution to host protein synthesis (36). Besides, Trp and its metabolites perform a crucial roles in different physiological processes, as a protein- building molecule to maintain cell growth and, simultaneously coordinating the body’s response to the environment, as a neurotransmitter and signal molecule (37).

**Figure 1:**
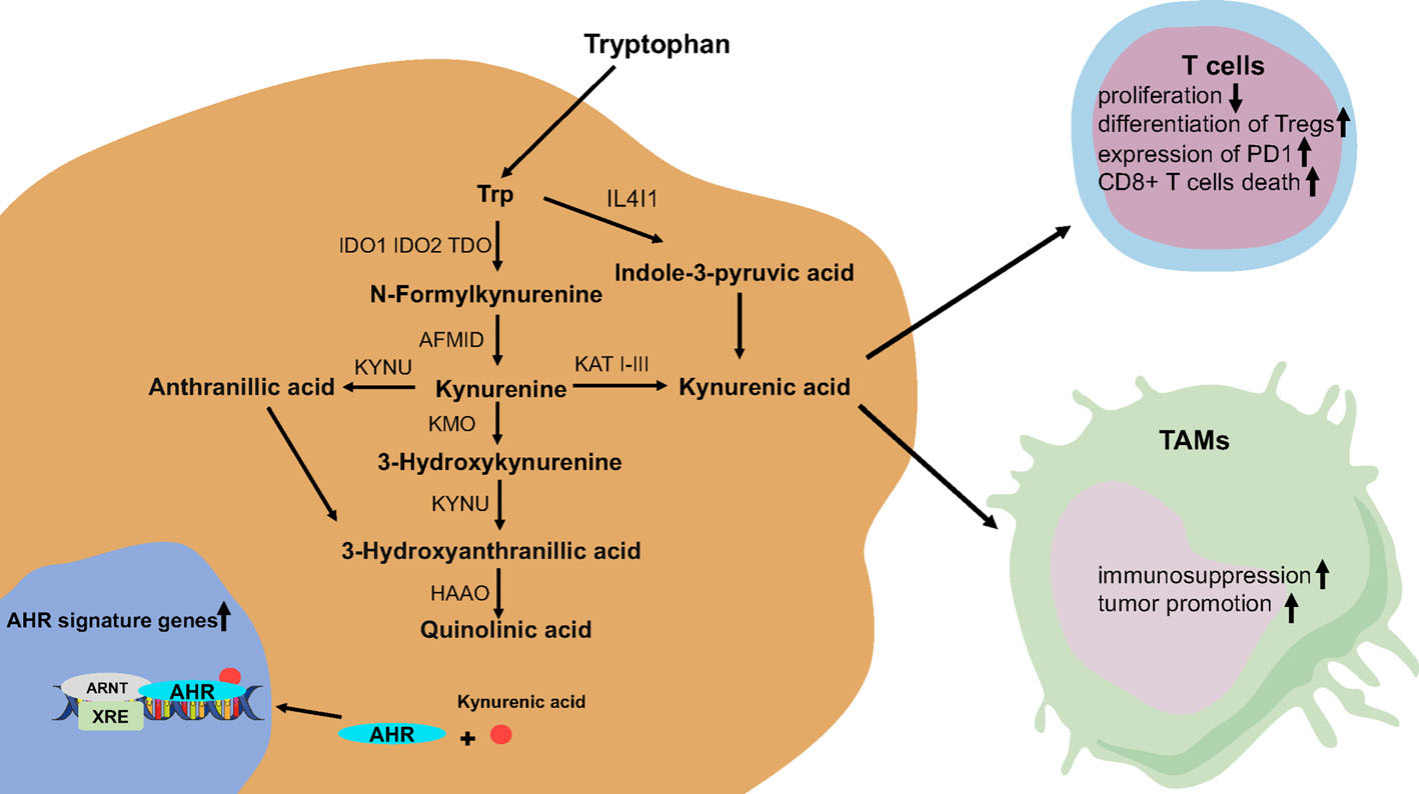
The tryptophan catabolic pathway in glioma. AFMID, kynurenine formamidase; AHR, aryl hydrocarbon receptor; HAAO, 3-hydroxyanthranilate 3,4-dioxygenase; IDO, indoleamine-2,3-dioxygenase; KATs, kynurenine amino transferases I–III; KMO, kynurenine 3-monooxygenase; KYNU, kynureninase; TDO, tryptophan-2,3-dioxygenase.

The activity of Trp metabolic pathways determines the level of free Trp. There are three pathways of tryptophan catabolism including (1): tryptamine is formed after decarboxylation by aromatic l-amino acid decarboxylase (AADC) (2); formation of 5-hydroxytryptamine (5-HT) by tryptophan hydroxylase (TPH) (3); kynurenine (Kyn) pathway (KP) is the most critical way of Trp degradation; more than 95% of free Trp is a substrate of the KP for Trp degradation (36, 38, 39). Interestingly, indoleamine-2,3-dioxygenase 1/2 (IDO1/IDO2) and tryptophan-2,3-dioxygenase (TDO) are major rate-limiting enzymes, which catalyze the oxidative cleavage of the indole ring of Trp in the KP (40). At the same time, kynurenase (KYNU), kynurenine aminotransferase (KATI KATIII), Kynurenine monooxygenase (KMO), and other enzymes participate in the KP and produce a variety of metabolites, including neuroactive and neurotoxic substances (anthranilic acid (AA) and quinolinic acid (QA)), which affects the growth and function of human cells (41) (42) (43) (**Figure 1**).

## The Reprogramming of Tryptophan Metabolism in Glioma

Generally, the expression level of KP enzyme and metabolites in different cells and tissues is strictly regulated, but the abnormality of Trp metabolism and the accumulation of metabolites to varying degrees are involved in a wide range of disease processes, including tumors, neurodegenerative diseases, and self-immunity and mental illness, etc. Moreover, dysfunction of KP enzymes can trigger or facilitate diseases of CNS (41). For example, tryptophan tryptophan metabolites were found to accumulate in large amounts in the cerebrospinal fluid of glioma patients (44). Conversely, the enhanced activity of enzymes such as IDO, which are highly expressed in glioma cells, leads to the decrease of tryptophan and the accumulation of metabolites in the cells and microenvironment. Finally, these two promote the growth and invasion of gliomas, and meanwhile inhibit the anti-tumor immune response in the microenvironment (45).

**Figure 2:**
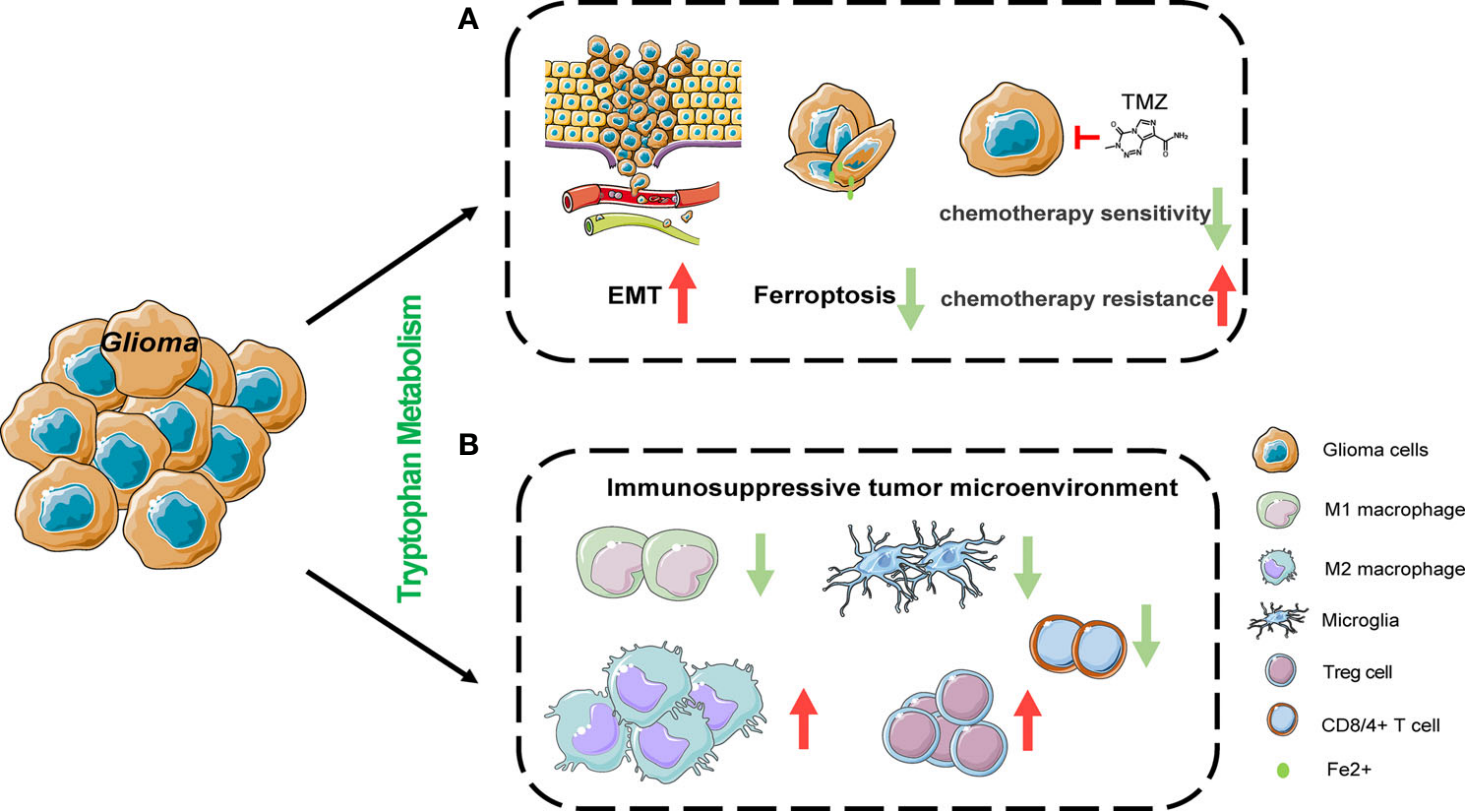
The effects of tryptophan metabolism in cancer cell and its microenvironment. **(A)** Tryptophan metabolism mediated by IDO1 and TDO could promote the migration and invasion of glioma cells *via* Kyn/AHR signaling pathway. IL4I1 catabolizes tryptophan into indole-3-pyruvate (I3P) to inhibit ferroptosis by expressing anti-oxidative gene expression program in Hela cells. IDO1 is induced as an undesired effect of chemotherapy in non-small-cell lung cancer and breast cancer. **(B)** Tryptophan metabolism suppresses T cell proliferation and function by inducing the differentiation of regulatory T cells (Tregs), the expression of programmed cell death protein 1 (PD1) on CD8+ T cells, cell death of CD8+ T cells and the recruitment of immunosuppressive tumor-associated macrophages. EMT, epithelial-mesenchymal transition.

Studies have revealed that changes in tryptophan metabolism can promote tumor development by enhancing the malignant characteristics of tumor cells and the immune suppression in the tumor microenvironment (46, 47). IDO1 is widely expressed in gliomas and is predictive of a poor prognosis in glioma patients. Likewise, it has also been established that TDO can promote tumor progression (47, 48). IDO1 and TDO, which are both positively related with the glioma grade, could promote the migration and invasion of glioma cells *via* the Kyn/AHR/APQ4 signaling pathway (49).The IDO1 expression level is elevated in glioma stem cells compared to GBM cells, and IDO1 leads to therapeutic resistance through the promotion of immunosuppression (50). More recently, interleukin-4-induced-1 (IL4I1) has been demonstrated to be more closely associated with AHR activity than IDO1 or TDO2 and is defined as the main Trp-catabolic enzymes in GBM (51). Furthermore, IL4I1 catabolizes tryptophan into indole-3-pyruvate (I3P) to inhibit ferroptosis by expressing an anti-oxidative gene expression program (52). Accumulation of the neurotoxic substance QA has also been found in gliomas. However, some studies revealed that in the glioma tumor microenvironment, QA is mainly produced in microglia rather than tumor cells (6, 53). The relationship between QA and the pathophysiological process of glioma warrants further studies. More importantly, the role of tryptophan and its metabolites in the immune microenvironment of glioma cannot be overlooked, and new immunotherapy targets may be discovered regarding its regulatory mechanisms (**Figure 2**).

## The Effect of Tryptophan Metabolism on the Immune Microenvironment of Glioma

### The Distribution of GAMs

Present-day research has found that GAMs control the onset, progression, metastasis, and response to treatment of glioma (54, 55). Enhancing the immunosuppression of the tumor microenvironment is considered a potential mechanism of GAMs to promote cancer. The phenotype M2-like macrophages of GAMs is associated with progression and immunosuppression in glioma (56). M1-type macrophages mainly utilize aerobic glycolysis during activation, which is associated with increased glucose uptake and the conversion of pyruvate to lactic acid. Nevertheless, the energy of M2-type macrophages is derived from fatty acid oxidative metabolism (57). The blockage of lipid metabolism may not only block M2 polarization, but also drive macrophages to return to the M1 state (58). The tryptophan metabolite Kyn produced by glioma cells participates in its function by activating the AHR in GAMs (59). The AHR-dependent transcription program regulates the recruitment and activation of GAMs in GBM. AHR can upregulate the expression of chemokine (C-C motif) receptor 2 (CCR2) of GAMs, and further drive GAM recruitment and aggregation (28). A study has found, in gliomas, AHR participates in the key pathways that regulate the polarization of macrophages in glioma by inhibiting NF-κB signaling and promotes the anti-inflammatory response driven by KLF4 (60).

At the same time, there may be multiple mechanisms regulating GAMs activation and infiltration in GBM. It was found that CSF1R signaling can alter the phenotype of GAMs (61). Moreover, the inactivation of NF1 in MES-GBM promotes the infiltration of GAMs. MES-GBM showed higher infiltration of GAMs than other subtypes, consistent with the high expression of CCL2 and CCR2 (62). Since the expression of AHR in MES-GBM is not higher than that in other subtypes, these findings indicate that AHR is one of several mechanisms in GBM that control GAMs recruitment and functions through mediators such as CCL2 and CCR2.

In conclusion, AHR activated during tryptophan metabolism participates in the mechanism of GBM controlling GAMs.

## The Differentiation of Regulatory T Cells (Treg)

Treg is defined as a functional subset of suppressor T cells, mainly produced by the differentiation of CD4+ T cells induced by cytokines *in vivo*. Immunosuppression and immune incompetence are two crucial functional characteristics of Treg cells. The Trp-Kyn-AHR pathway is known to play a significant role in the differentiation process of Treg cells. The increased activity of rate-limiting enzymes such as IDO leads to the continuous consumption of tryptophan in the microenvironment. This relatively hungry environment causes the surrounding T cell cycle to stagnate, facilitating Treg production (63). Some studies also believe that the activation of the KP leads to the accumulation of Kyn in the microenvironment. Kyn activates AHR on CD4+ T cells through classic response genes such as Cyp1a1 and Cyp1b1, thereby inducing CD4+ T cells to differentiate into Tregs (44). Thus, Treg cells inhibit the function of effector T cells and regulate the body’s immunity by secreting inhibitory cytokines or interacting with antigen-presenting cells. The differentiation of IL-10 producing Tr1 cells can be promoted by AHR (64, 65) contribute to tumor-associated immunosuppression together with Foxp3+ Tregs (32).

A previous study noted that M2-type polarized microglia could induce Tregs production *in vitro*, highlighting the regulatory role of microglia in adaptive immune response and demonstrating the indirect regulatory role of AHR on Tregs (66).

## T Cell Anergy or Death

Due to the increased expression and activity of rate-limiting enzymes such as IDO and TDO, and the activation of KP, the loss of tryptophan in the tumor microenvironment increases, which activates the amino acid starvation stress response, such as the expression of protein kinase GCN2 in surrounding cells (67). Protein kinase GCN2 is a vital regulator of the integrated stress response (ISR), regulating protein synthesis when amino acids are scarce. Studies have found that T cells with high GCN2 expression appear anergic and induce cell death (68). After the knockout of the GCN2 gene by antigen-presenting cells (APCs), autophagy decreases and amount of reactive oxygen species increase, which in turn activates the production of inflammasomes and IL-1β, leading to increased inflammation and helper T lymphocytes 17 (Th17) response. Therefore, local depletion of tryptophan is considered to be the principal “starvation and death” mechanism of immunosuppression. Other than the depletion of tryptophan, the activation of KP leads to the accumulation of tryptophan metabolites in the tumor microenvironment. These metabolites may individually or synergistically cause T cell anergy or death (14). The Trp-Kyn-AHR pathway executes an essential role in the regulation of T cell immunity in cancer. After a high level of Kyn is released into the tumor microenvironment, it is transferred to adjacent CD8^+^ T cells through transporters such as SLC7A8 and PAT4 (69). Besides, AHR is activated and increased by Kyn in T cells, thereby upregulating the expression level of PD-1, inhibiting T cell activity, and promoting immune tolerance. The expression of CD39 is promoted by AHR in GAMs, which promotes CD8^+^ T cell dysfunction by cooperating with CD73 to produce adenosine in the immune microenvironment of glioma (70). Apart from IDO and TDO, IL4I1, a secreted L-phenylalanine oxidase, has recently been defined as a novel immune checkpoint, playing a paramount role in Trp catabolism (71). IL4I1 promotes glioma cell migration *via* the Kyn/AHR pathway, and suppresses T cell proliferation in glioblastoma, which is associated with poor survival of glioma patients (51). Kyn can induce selective apoptosis of mouse thymocytes and Th1-cells, but cannot induce apoptosis of Th2-cells (72). GBM cells overexpress TDO2 to suppress T cell proliferation under standard oxygen conditions, however, the proliferation of the T cells could recover because of the reduction in kynurenine levels produced by the GBM cells (73). The immunomodulatory effect of Kyn on different lymphocyte subsets may be necessary for maintaining the homeostasis of peripheral lymphocytes and the accumulation of autologous lymphocytes.

GBM produces an angiogenic and inflammatory microenvironment that results in increased expression of adhesion molecules on endothelial cells and decreased tight junctions, leading to the formation of a highly permeable blood-brain barrier (BBB).These changes will enable more peripheral lymphocytes to participate in the immune microenvironment of the tumor. Nevertheless, GAMs remain the most essential part of the immune regulation of glioma.

## The Regulation of Antigen-Presenting Cells (APCs) and Myeloid-Derived Suppressor Cells (MDSCs)

Studies have found that after activation of kyn, AHR can participate in regulating the function of dendritic cells (DC) and promote the differentiation of effector T cells and regulatory T cells (74). Moreover, AHR can induce DCs to produce kyn and retinoic acid (RA), and promote the differentiation of Tregs (75). Furthermore, AHR also inhibits the activation of NF-kB in DCs through a mechanism mediated by SOCS2 (76), thereby interfering with the production of cytokines that promote the differentiation of effector T cells.

The loss of the BBB integrity allows MDSCs to infiltration in GBM, and these cells actively inhibit immune responses through PDL1 and CTLA4 (77). MDSCs have been considered as a prime mediator in the tumor microenvironment, delivering a powerful immunosuppressive effect on T cells (78). IDO overexpressed in tumors recruits and activates MDSCs through a Treg-dependent mechanism to exert its immunosuppressive effect. At the same time, studies have found that IDO is highly induced in tumor-invasive MDSCs (78, 79). These results indicate that IDO has functional diversity in immune evasion related to MDSCs.

In addition, AHR can participate in the regulation of the activities of astrocytes and microglia, which play a substantial role in the immune activities of the tumor microenvironment (80). Therefore, the signaling pathways induced by KYN-AHR participate in the regulation of GAMs and other immune cells. Identifying of common cell-specific mechanisms, including tumor metabolites and other molecules in the tumor microenvironment, can guide therapies for GAMs and other immune cells.

## Therapeutic Strategies for Immune Checkpoints

Recent researches demonstrate a series of therapy which set aberrant tryptophan metabolism as a target to improve the effects of glioma therapy. The Kyn/AHR pathway is considered as a significant factor in radiotherapy-induced immune checkpoint reactivation. Radiotherapy response can be enhanced by GDC-0919, known as IDO1 inhibitor, with the ability to pass through BBB and reduces radiotherapy-induced immunosuppression (81). 1-MT, an IDO1 inhibitor, added with two-fraction radiotherapy significantly reduced tumor size and increased survival in bears with GBM compared to untreated controls (82). These findings provide novel insights to enhance the effect of radiotherapy in glioblastoma. In chemotherapy, PCC0208009, a highly effective IDO inhibitor, combined with TMZ, enhanced the chemotherapy by promoting the immune response *in vivo*, including the percentages of CD3+, CD4+ and CD8+ up-regulated T cells. This result indicates that the combination of IDO inhibitor-based immunotherapy with chemotherapy is a potential strategy for brain tumor treatment (83). Lately, inhibitors that specifically target tryptophan metabolism have been involved in the clinical study of glioma. PF-06840003 is a highly effective IDO1 inhibitor with antitumor effects. Up to 500mg BID was generally well tolerated. Long-term clinical benefits can be gained by a subset of patients with recurrent malignant glioma (84). All these findings support the hypothesis that targeting Trp metabolism can consolidate immune therapy and provide a novel insight for antitumor treatment. At present, there are various researches on targeted drugs in the metabolism of tryptophan in gliomas, as shown in **Table 1**.

**Table 1:** Drug research aimed at key targets in the tryptophan metabolic pathway in glioma.

Drug	Indication		Comments	Clinical phase	
IDO pathway inhibitors					
Indoximod (D-1-MT)	GBM, glioma, gliosarcoma		Does not inhibit IDO1 *in vitro* Combined with temozolomide, bevacizumab and radiation	Phase I/II	
PCC0208009	Solid tumors		Combined with temozolomide	Experimental	
PF-06840003	Oligodendroglioma, astrocytoma, malignant		Noncompetitive kinetics with Trp Central nervous system penetration As single agent	Phase I
BMS-986205	Glioblastoma		Irreversible inhibitor Binds to heme-free apo IDO1 Combined with Nivolumab, Radiation Therapy, Temozolomide	Phase I
INCBO24360	Glioblastoma		Trp-competitive inhibitor Combined with Nivolumab	Phase III
GDC-0919	Solid tumors		Based on 4-phenylimidazole scaffold As single agent	Phase I
NLG802	Solid tumor		Prodrug of indoximod As single agent	Phase I
SHR9146+SHR- 1210	Solid tumor		Combined with apatinib	Phase I
MK-7162	Solid tumor		Combined with pembrolizumab	Phase I
1-Methyl-l-tryptophan (L-1-MT)	Solid Tumors		Trp-competitive inhibitor	Experimental
KHK2455	Solid Tumors		Binds to heme-free apo IDO1 As single agent	Phase I
LY3381916	Solid Tumors		Binds to newly synthesized apo-IDO1 but does not inhibit mature heme-bound IDO1 As single agent	Phase I
TDO inhibitors				
680C91	Solid Tumors		Nanomolar activity *in vitro* Low aqueous solubility Poor oral bioavailability	Experimental
LM10	Solid Tumors		Investigated in mouse cancer model	Experimental
4-(4-fluoropyrazol-1-yl)-1,2-oxazol-5-amine	Solid Tumors		Nanomolar cellular activity Sixfold selectivity over IDO1	Experimental
Fused imidazo-indoles	Solid Tumors		TDO selective	Experimental
Indazoles	Solid Tumors		TDO selective	Experimental
Dual IDO1–TDO inhibitors				
DN1406131	Advanced Solid Tumors		As single agent	Phase I
HTI-1090	Solid Tumors		Potent, orally bioavailable dual IDO1/TDO inhibitor	Phase I
RG70099	Solid Tumors		Significantly reduces Kyn levels in preclinical tumor models	Experimental
EPL-1410	Solid Tumors		Reduces tumor volume and Kyn : Trp ratio in cancer models	Experimental
AHR antagonists				
CB7993113	Solid Tumors		Identified by ligand-shape-based virtual screening	Experimental

IDO, indoleamine 2,3 dioxygenase; TDO, tryptophan-2,3- dioxygenase; Trp, l-tryptophan; AHR, aryl hydrocarbon receptor.

In the existing immune cell therapy, activating CD4+ and CD8+ T cells would increase the risk of autoimmune diseases if all Tregs are eliminated. Therefore, only part of Treg cells can be eliminated before the T cells are returned to the patient. The use of Treg cell-specific antibodies to eliminate the effect of Treg on immunosuppression is worthy of consideration.

## Conclusions

The changes in tryptophan metabolism in glioma lead to a series of alterations in tumor cells and the surrounding tumor microenvironment. These metabolic variations may enable glioma to evade the immune system by affecting tumor cells and immune microenvironment and further promote tumor progression. Consequently, research on new metabolic, immune checkpoints can strengthen the effect of immunotherapy in the future. This will open up a new era of comprehensive treatment of glioma.

## Author Contributions

YX and HZ wrote the article. QS and RG drew the figures and table. FY polished the language. BL and QC supervised the article. All authors contributed to the article and approved the submitted version.

## Funding

This work was supported by the National Natural Science Foundation of China (No.82072764).

## Conflict of Interest

The authors declare that the research was conducted in the absence of any commercial or financial relationships that could be construed as a potential conflict of interest.

## Publisher’s Note

All claims expressed in this article are solely those of the authors and do not necessarily represent those of their affiliated organizations, or those of the publisher, the editors and the reviewers. Any product that may be evaluated in this article, or claim that may be made by its manufacturer, is not guaranteed or endorsed by the publisher.

## Abbreviation

AADC, amino acid decarboxylase; DC, dendritic cell; IDO-1, Indoleamine 2,3-dioxygenase 1; IDO2, indoleamine 2,3-dioxygenase 2; TDO, tryptophan 2,3-dioxygenase; Trp, L-tryptophan; Kyn, L-kynurenine; QA, quinolinic acid; AHR, aryl hydrocarbon receptor; 5-HT, 5-hydroxytryptamine; GCN2, general control nonderepressible 2; GAM, Glioma-associated macrophage; GSCs, Glioma stem cells; LAT, large amino acid transport; GSH, glutathione; GS, glutamine synthetase; APC, antigen-presenting cell; MDSC, myeloid-derived suppressor cell.
